# Cardiorespiratory Fitness as a Mediator of the Influence of Diet on Obesity in Children

**DOI:** 10.3390/nu10030358

**Published:** 2018-03-16

**Authors:** Noelia Lahoz-García, Antonio García-Hermoso, Marta Milla-Tobarra, Ana Díez-Fernández, Alba Soriano-Cano, Vicente Martínez-Vizcaíno

**Affiliations:** 1 Centro de Estudios Socio-Sanitarios, Universidad de Castilla-La Mancha, 16071 Cuenca, Spain; noelia.lahoz@alu.uclm.es (N.L.-G.); mmilla@gmail.com (M.M.-T.); alba.soriano@uclm.es (A.S.-C.); Vicente.martinez@uclm.es (V.M.-V.); 2 Laboratorio de Ciencias de la Actividad Física, el Deporte y la Salud, Facultad de Ciencias Médicas, Universidad de Santiago de Chile, USACH, Santiago 7500618, Chile; antonio.garcia.h@usach.cl; 3 SESCAM, Department of Nutrition and Dietetics, Talavera de la Reina, 45600 Toledo, Spain; 4 Facultad de Enfermería, Universidad de Castilla-La Mancha, 16071 Cuenca, Spain; 5 Facultad de Ciencias de la Salud, Universidad Autónoma de Chile, Talca 1670, Chile

**Keywords:** children, obesity, energy intake, fat intake, CRF, mediation

## Abstract

The association between diet and obesity has been widely studied and it continues to be controversial; however, the extent to which cardiorespiratory fitness (CRF) acts as a confounder or mediator in this relation has not been analyzed. The aim of this study is to examine if the relation between diet and obesity is mediated by CRF. In this cross-sectional study, fat mass (by electronic bioimpedance) was measured in 320 schoolchildren, aged 9–11 years. Diet was measured through two computerised 24-h dietary recalls and CRF was assessed by the 20-m shuttle run test. Simple mediation analyses were fitted. CRF acts as a partial mediator in the negative relationship between dietary factors (energy intake/weight, carbohydrate intake/weight, protein intake/weight, and fat intake/weight) and fat mass. The percentage of mediation ranged from 24.3 to 33.2%. Thus, Spanish schoolchildren with higher levels of energy and macronutrients intake had lower adiposity levels, especially when they had good levels of CRF.

## 1. Introduction

Childhood obesity has reached epidemic proportions worldwide [1,2,3]. In Spain, more than 35% of children are overweight or obese [4], similar to rates in other European countries [5] and the obesity prevalence found in the United States [1]. Obesity confers both adverse health consequences during childhood and long-term effects [1,2], which highlights the importance of studying obesity from childhood.

Obesity occurs when energy intake exceeds energy expenditure. The development of obesity is mediated by multiple genetics that influence the susceptibility of a given child to become obese, and environmental factors, which seem to play major roles in obesity development [3,6]. Diet and cardiorespiratory fitness (CRF), in spite of the genetic component of CRF [7], are some of these environmental factors [5,8]. The relation between dietary factors and obesity in children has been widely examined, and some cross-sectional studies have shown a negative association between total energy intake (EI) and fat mass [9,10,11]; however, these findings have not been constant, and while some studies in the literature have shown positive relations [12], others have found no relation [13].

Evidence from several reviews [6,7,14] has demonstrated a strong negative correlation between CRF and overweightness in children and adolescents in most of the studies included. Additionally, changes in CRF following exercise training have been found by other reviews; however, there were few randomised controlled trials included in them [15]. An effect of physical activity training in overweight and obese adolescents aged 12–17 years has also been reported, where improvements in fitness were found in eight of the 11 studies included, and no relations were found in the rest [16].

However, there is a lack of evidence regarding the association between CRF and diet factors, both key variables for maintaining the energy balance.

Mediation analysis allows us to estimate the association between two factors, and the extent to which a third variable could modify, mediate, or confound the association. Due to the controversial relation found in the literature between diet, cardiorespiratory fitness, and obesity in children, and the reported relationship between CRF and body fat, we hypothesized that CRF could act as a mediator in the relationship between diet and obesity. To our knowledge, no study has examined if the relationship between diet and obesity in children is attenuated or mediated by CRF. The aim of this study was twofold: first, to assess the association of different components of diet with body composition variables in 9–11 year-old Spanish children, and second, to explore the mediatory influence of CRF on this association.

## 2. Materials and Methods

### 2.1. Design

A cross-sectional analysis was conducted of the baseline measurements (September-November 2010) of a cluster-randomised trial (registered at Clinicaltrials.gov, number NCT01277224). The Clinical Research Ethics Committee of the ‘Virgen de la Luz’ Hospital in Cuenca, as well as the Director and Board of Governors (Consejo Escolar) of participating schools, approved the study protocol. Written consent was provided by the parents for the participation of their children. A total of 320 fifth graders, 9–11 years old, from public schools in the province of Cuenca, Spain, were included.

The protocol explained that participants completed most of the variables assessment in the first session (anthropometric variables, CRF and the first of the two 24-h dietary recall). In a second session (another day in the same week), only a second 24-h dietary recall was measured. More information has been reported previously [17].

### 2.2. Measures

#### 2.2.1. Anthropometrics

Participants were weighed twice whilst wearing light clothing, using a digital scale (SECA model 861; Vogel & Halke, Hamburg, Germany) with an accuracy of 100 g. Participants’ height was measured twice without shoes, using a wall-mounted stadiometer (SECA 222) to the nearest 0.1 cm. The mean of each of these two measurements was used to calculate BMI (kg/m^2^). Fat mass (FM), expressed as the percentage of body fat, was estimated using a bioimpedance analysis system (BC-418, Tanita Corp, Tokyo, Japan). Bioimpedance analysis by the BC-418 Tanita system had showed a good correlation coefficient (*r* = −0.87) with the reference method, dual energy X-ray absorptiometry (DXA), in %fat estimate for total body [18]. Percentage of body fat was used as the most accurate estimation of adiposity since its use in population-based studies has been repeatedly recommended [19]. Waist circumference (WC) was defined as the average of two measurements taken with flexible tape at the midpoint between the last rib and the iliac crest. Finally, the fat mass index (FMI = fat mass/height^2^—in kg/m^2^) was calculated. This index has been widely used in studies in children. It allows for a more exact comparison between fatness and BMI, since they are both computed and expressed by dividing fat mass and weight by squared height in metres. A recent study showed that FMI is a stronger predictor of cardiovascular mortality than FM [20]. Sexual maturation was assessed by parents using figures depicting the Tanner stages of sexual maturity. Children were classified as underweight, normal weight, overweight, or obese according to sex- and age-specific BMI cut offs defined by Cole and Lobstein [21]. All physical examinations were performed by trained fieldworkers.

#### 2.2.2. Dietary Factors

Food consumption was measured twice, once with respect to a weekday and once with respect to a weekend day, using a self-administered computerised 24-h dietary recall, the Young Adolescents’ Nutrition Assessment on Computer (YANA-C). The YANA-C was completed autonomously by participants, with two or three research members on hand to provide assistance if required, and to check, when children had completed the questionnaire, in the overview screen extreme values or missing data such as intake of beverages, bread, sauces, etc. The YANA-C has been previously shown to have moderate to good levels of reliability and validity [22]. Total daily EI in kcal and total daily macronutrient intake in grams were obtained from the weighted average of the two 24-h recalls. We estimated the habitual EI and macronutrients intake considering that the week day 24-h recall contributed to 5/7 of the habitual diet, and the 24-h weekend recall to 2/7 of it. Macronutrients (g) relative to the children´s weight (kg) were calculated (as WHO express energy and protein requirements); each macronutrient intake was also expressed as a percentage of kcal over the total EI. The compositions of all food and beverages were estimated using the food composition tables produced by the Centre for Superior Studies in Nutrition and Dietetics (CESNID) [23].

#### 2.2.3. Cardiorespiratory Fitness

CRF was assessed by the 20-m shuttle run test, in which participants have to run between two lines 20-m apart, while keeping pace with audio signals emitted from a pre-recorded compact disc. The initial speed was 8.5 kmh^−1^, and this was increased by 0.5 kmh^−1^ min^−1^ (stage duration = 1 min). The test finished when the child failed to reach the end line at the same time as the audio signal, and the last half stage completed (1; 1.5; 2; 2.5 ...) was recorded as an indicator of children´s CRF. VO_2_ max (mL/kg/min) was calculated using the Léger protocol [24].

### 2.3. Statistical Analysis

The distribution of continuous variables was checked for normality before the analysis. Sex differences in descriptive data were assessed by the Student’s *t*-test for continuous variables and Pearson χ^2^ for categorical variables.

Partial correlation coefficients were estimated to examine the relationship between body composition variables, CRF, and dietary factors, controlling for age and sex. The variables CRF, EI/weight, carbohydrate/weight, protein/weight, and fat/weight were categorized as low (first quartile), medium (second and third quartiles), or high (fourth quartile). ANCOVA models were fitted to test differences in FM by categories of CRF and dietary factors, controlling for age and sex (model 1), and with further adjustment for EI/weight, carbohydrate/weight, protein/weight, fat/weight, or CRF, depending on the fixed factor (models 2–6). Pairwise post-hoc hypotheses were tested using the Bonferroni correction for multiple comparisons.

To examine whether the association between dietary factors and obesity was mediated by CRF, a mediation analysis was conducted using the PROCESS macro for SPSS (SPSS, Inc., Chicago, IL, USA) [25]. The goal of this model was to investigate the total (c) and direct effects (a, b, c’), which indicates the unstandardized regression coefficient, and significance between the independent and dependent variables in each model. It also investigates the indirect effect (IE), obtained from the product of coefficients (a * b), which shows the change in the body composition variable for every unit change in the dietary factor that is mediated by CRF. This macro used bootstrapping methods as recommended by Preacher and Hayes [26] for testing mediation hypotheses, using a resample procedure of 10,000 bootstrap samples. Point estimates and confidence intervals (95%) were estimated for the IE. The point estimate was considered to be significant when the confidence interval did not contain zero. Mediation was also assessed using the steps outlined by Sobel [27]: first, the IE was estimated, and then divided by its standard error, and a *Z* test of the null hypothesis where the IE is equal to zero was performed. Finally, the percentage of mediation was calculated with the ratio of the indirect (a * b) to the total effect (c).

A bilateral criterion for statistical significance of *p* ≤ 0.05 was used. All statistical analyses were performed using the software IBM SPSS 23 (SPSS, Inc., Chicago, IL, USA).

## 3. Results

### 3.1. Descriptive Statistics

Table 1 shows the characteristics of the study population. Girls were significantly taller than boys (*p* = 0.003) and they had a higher percentage of fat mass than boys (*p* < 0.001). CRF performance was better among boys (*p* < 0.001). The protein/weight ratio was significantly higher in boys than in girls.

Partial correlation coefficients between body composition variables and dietary factors, controlling for age and sex, are shown in Table 2. All body composition variables (BMI, WC, FM, and FMI) and some dietary factors (EI/weight, carbohydrate/weight, protein/weight, and fat/weight) were negatively correlated (*p* < 0.01). Total EI was only associated with FM (*p* < 0.01). The percentage of energy from proteins was positively correlated with FM and WC (*p* < 0.05). Positive correlations were observed between CRF and dietary factors, while negative correlations were found between CRF and body composition variables (*p* < 0.001).

No changes in the direction or significance of partial correlation coefficients were found when examining the relationship between body composition variables and dietary factors controlling for CRF, age, and sex, and thus, CRF is likely not a confounding variable in the association between diet and obesity (data not shown).

Mean differences in FM according to CRF categories, controlling for age and sex (model 1), are shown in Table 3. FM was significantly worse in children with lower CRF. Similar results were obtained when dietary factors (EI/weight, carbohydrate/weight, protein/weight, and fat/weight) were included in the ANCOVA models as covariates (models 2–5). All Bonferroni post-hoc comparisons of means indicated statistically significant differences in FM by CRF categories (low > medium > high).

Mean differences in FM according to categories of dietary factors, controlling for age and sex (model 1), are shown in Table 4. FM was significantly higher in children with a lower EI/weight ratio; in the same way, children with a lower intake of carbohydrates/weight, protein/weight, and fat/weight had a significantly higher FM than children with a higher macronutrients intake. Similar results were obtained when CRF was added to the ANCOVA models as a covariate (model 6). Pairwise mean comparisons using the Bonferroni post-hoc test were statistically significant for categories of dietary factors (low > medium > high). ANCOVA models fitted with other body composition variables measured (BMI, WC, and FMI), instead of FM, showed a similar result (Supplementary Materials Tables S1 and S2).

### 3.2. Simple Mediation Analysis

**Figure 1:**
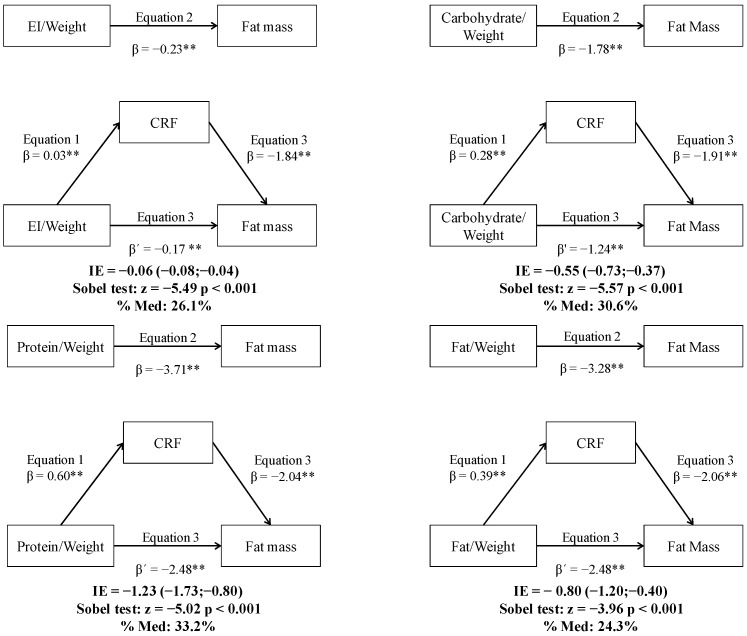
Simple mediation models of the relationship between dietary factors and fat mass, using CRF as a mediator, controlling for age. EI, energy intake; CRF, cardiorespiratory fitness; IE, indirect effect; %Med, percentage mediated by fat mass. ** *p* < 0.001.

Mediation analysis diagrams are depicted in Figure 1. Overall, CRF acted as a partial mediator in the relationship between dietary factors (EI/weight, carbohydrate/weight, protein/weight, and fat/weight) and FM. In the first regression step (Equation (1)), the dietary factor was positively related with CRF (*p* < 0.001). In Equation (2), the regression coefficient of the dietary factor on the dependent variable, FM, was significant (*p* < 0.001). In the last regression model, CRF was negatively related to FM (*p* < 0.001), but when both the dietary factor and CRF were included in the model, though the regression coefficient decreased, it remained statistically significant. To confirm the mediator role of CRF, both IE (range from −0.06 to −1.23, not including zero in the 95% CI) and the Sobel test (*p* < 0.001) were significant. The percentage of the total effect mediated by CRF in the relationship between dietary factors and FM ranged from 24.3% and 33.2%. The result of the mediation analysis between CRF, diet, and other adiposity variables measured (BMI, WC, and FMI) showed similar mediation effects (Supplementary Materials Figures S1–S3).

## 4. Discussion

### 4.1. Statement of Principal Findings

The relation between obesity and diet has been extensively analyzed, but the effect of CRF as a mediator has not been studied. The present study is, to our knowledge, the first to evaluate the mediating role of CRF in the relation between dietary factors and adiposity in children. Our data show that children in the lowest CRF category have higher FM than children in higher categories of CRF. Similarly, children with the lowest EI/weight, carbohydrate intake/weight, protein intake/weight, and fat intake/weight showed the highest FM compared to children with higher intakes. Additionally, our data showed that CRF is a partial mediator in the relation between energy and macronutrient intakes with obesity.

### 4.2. Direction of the Association between CRF and Obesity

The relationship between CRF and obesity is well known. Some systematic reviews have reported a consistently negative association between CRF and excess body fat [6,7,14] measured as BMI or body fatness. Accordingly, our data show that CRF is negatively associated with FM, as a percentage of body fat, in Spanish schoolchildren.

### 4.3. Direction of the Association between Diet and Obesity

Several studies have tried to elucidate the relation between diet and adiposity; however, this relation continues to be controversial [28]. Our results showed a negative association between total EI and FM. These results are in line with some previous studies, which found a negative association between EI and obesity [9,10,11]. However, no relationship between these two variables [13], and even a positive association between them, have also been reported [12,29]; though these studies measured neither fitness nor physical activity (self-reported or objective), which could explain the difference with our results. Our data also reflect that macronutrients intake ratios were negatively associated with adiposity variables.

### 4.4. Direction of the Association between CRF and Diet

Few studies have investigated the relation between dietary factors and CRF in children or adolescents. Our data showed a positive association between total EI and CRF. This finding is in line with previous research, which found a positive association, only in boys, between total EI and CRF with similar analytical techniques [30]. Another study, including young men and women, found no relation between EI by CRF tertiles [31], probably because of its small sample size.

In our study, children with better CRF reported a higher intake of carbohydrates, proteins, and fats. Previous studies found better CRF in children who had medium or high adherence to the healthy Mediterranean diet [32], and children with a high frequency of fruits and vegetables intake [33], but only food group relationships, not macronutrients intake, were investigated in these studies.

### 4.5. CRF as a Mediator between Diet and Obesity

Our mediation analysis offers new insight into understanding the relationship between CRF, diet, and obesity, suggesting that the diet/obesity relationship is partially mediated by CRF. This supports the hypothesis that children with medium or high levels of CRF, and elevated intakes of energy and fat, are less prone to be obese than children with lower CRF and a lower intake of energy and fat.

The mediator role of CRF between cardiometabolic risk and adiposity has been demonstrated, since CRF attenuates the cardiometabolic risk scores in obese children [34]. Another study showed that upgraded CRF is more effective than weight loss to improve the metabolic profile of obese children [35]. The positive effect of healthy dietary habits over fitness was previously tested isolated [30,33] and linked to an exercise training program in obese children [36]. Some of these studies have also exhibited an improvement in CRF due to the reduction of BMI [35,36], which might show the mediator effect of CRF on the relationship between diet and obesity.

The results of our study provide further evidence supporting that the effect of diet on obesity is partially mediated by fitness in 9 to 11 year old Spanish children. This means we can expect improvements in adiposity levels in obese children associated with interventions aimed at improving fitness with no changes in dietary factors.

Since fitness has been shown in several reviews to be very related to high-intensity physical activity [15,16,37], our results could be partially explained by the theory proposed by Gutin [38], in which the amount of physical activity by young children is a potent predictive factor of their fat and lean tissue. Physical activity stimulates physiologic processes that conduct nutrient intake to be partitioned in muscle and bone instead of adipose tissue. Moreover, physical activity during early childhood improves CRF, which mediates the relation between diet and obesity. This allows active children to ingest more energy and fat, which is essential for adequate growth, without becoming obese.

### 4.6. Limitations

The main limitation of our study is its cross-sectional design, which prevents us from making causal inferences. Prospective studies could confirm or broaden our results. Secondly, the use of self-reported dietary data could suffer from underreporting or unintentional measurement errors, especially in children [30]. To minimise this, a research member reviewed the 24-h dietary recalls before they were finished. Additionally, the use of a computer-assisted 24-h dietary recall has proven to be an appropriate method to measure dietary information in children [22]. Third, food group intake, not only macronutrients, needs to be measured in order to compare our results with previous studies. Fourth, we did not control for variance in sexual maturation. In our opinion, the homogeneity of the sample with respect to the Tanner stage made this unnecessary (Table 1). Moreover, not all of the children had the Tanner stages variable measured; thus, the control for this variable would decrease the statistical power of our study. Finally, the potential mediator was chosen based on previous evidence, and although CRF has been demonstrated to be a partial mediator in the relationship between diet and obesity, future follow-up studies testing multiple moderator/mediator models will be useful to clarify the potential mediator, moderator, or confounder role of others factors (i.e., amount of physical activity).

## 5. Conclusions

Our findings support that energy and fat intake are negatively associated with obesity, and that this relation is partially mediated by CRF. This is important from a public health point of view because they indicate that diet modifications could result in higher weight loss if they were associated with actions aimed at improving fitness in children.

## Figures and Tables

**Table 1:** Characteristics of the study sample.

	Boys (*n* = 164)	Girl (*n* = 174)	*p* ^a^
Age (years)	10.0 ± 0.5	10.0 ± 0.4	0.335
Weight (kg)	38.7 ± 8.7	39.9 ± 9.5	0.236
Height (cm)	140.6 ± 6.6	142.9 ± 7.0	0.003
BMI (kg/m^2^)	19.4 ± 3.6	19.4 ± 3.7	0.908
WC (cm)	68.9 ± 9.2	68.9 ± 9.3	0.978
FM (%)	24.1 ± 7.0	27.0 ± 5.7	<0.001
FMI (kg/m^2^)	4.9 ± 2.4	5.4 ± 2.2	<0.001
Tanner stage *	1.6 ± 0.7	1.6 ± 0.6	0.345
Tanner stage (I–II/III–V) (%) *	85.6/14.4	87.7/12.3	0.721
CRF (mL/kg/min)	44.33 ± 4.7	41.51 ± 3.3	<0.001
EI (kcal)	1648.1 ± 516.9	1582.0 ± 497.7	0.245
Carbohydrate (% EI)	42.0 ± 9.4	43.2 ± 9.6	0.251
Protein (% EI)	18.4 ± 4.9	17.6 ± 4.5	0.131
Fat (% EI)	39.7 ± 8.5	39.2 ± 8.7	0.611
EI/weight (kcal/kg)	45.1 ± 18.0	42.1 ± 16.8	0.132
Carbohydrate/weight (g/kg)	4.7 ± 2.0	4.5 ± 2.0	0.426
Protein/weight (g/kg)	2.0 ± 0.8	1.8 ± 0.8	0.042
Fat/weight (g/kg)	2.0 ± 1.1	1.9 ± 0.9	0.140
Underweight (%)	8.2	8.6	0.770
Normal weight (%)	54.1	55.7	
Overweight (%)	30.8	26.4	
Obese (%)	6.8	9.2	

BMI, body mass index; WC, waist circumference; FM, fat mass; FMI, fat mass index; CRF, cardiorespiratory fitness; EI, energy intake. The data are presented as mean ± s.d. ^a^ Gender group comparisons were conducted by *t*-test for continuous variables and Pearson’s χ^2^ for categorical variables: prevalence of Tanner stage and ponderal status. * All variables have complete data (i.e., *n* = 320), except for Tanner stage, which was available in 287 participants, 132 boys and 155 girls. Statistically significant values are presented in bold (*p* ≤ 0.05).

**Table 2:** Partial correlation coefficients among cardiorespitatory fitness, body composition variables, and dietary factors, controlling for age and sex.

	WC	FM	FMI	CRF	EI	Carbohydrate	Protein	Fat	EI/Weight	Carbohydrate/Weight	Protein/Weight	Fat/Weight
BMI	0.933 **	0.915 **	0.965 **	−0.571 **	−0.096	−0.036	0.119 *	−0.028	−0.583 **	−0.552 **	−0.469 **	−0.463 **
WC		0.906 **	0.928 **	−0.574 **	−0.088	−0.015	0.131 *	−0.056	−0.582 **	−0.547 **	−0.456 **	−0.470 **
FM			0.975 **	−0.602 **	−0.179 **	−0.005	0.156 *	−0.081	−0.604 **	−0.554 **	−0.462 **	−0.504 **
FMI				−0.584 **	−0.136 *	−0.025	0.135 *	−0.047	−0.578 **	−0.538 **	−0.454 **	−0.471 **
CRF					0.045	0.064	−0.048	−0.043	0.319 **	0.340 **	0.272 **	0.214 **
EI						−0.206 **	−0.207 **	0.341 **	0.806 **	0.625 **	0.611 **	0.776 **
Carbohydrate							−0.433 **	−0.871 **	−0.147 *	0.368 **	−0.383 **	−0.470 **
Protein								−0.066	−0.218 **	−0.405 **	0.394 **	−0.206 **
Fat									0.284 **	−0.185 **	0.212 **	0.634 **
EI/weight										0.838 **	0.781 **	0.900 **
Carbohydrate/weight											0.504 **	0.545 **
Protein/weight												0.693 **

BMI, body mass index; WC, waist circumference; FM, fat mass; FMI, fat mass index; CRF, cardiorespiratory fitness; EI, energy intake. * *p* < 0.05, ** *p* < 0.001.

**Table 3:** ANCOVA models comparing fat mass percentage by cardiorespiratory fitness categories in children.

FM (%)	Cardiorespiratory Fitness Categories			
	Low	Medium	High	*p*-Value
Model 1	31.8 ± 0.6	25.7 ± 0.4	21.0 ± 0.6	<0.001
Model 2	30.1 ± 0.6	25.8 ± 0.3	22.1 ± 0.5	<0.001
Model 3	30.5 ± 0.6	25.7 ± 0.4	22.0 ± 0.5	<0.001
Model 4	30.7 ± 0.6	25.7 ± 0.4	21.7 ± 0.5	<0.001
Model 5	30.6 ± 0.6	25.9 ± 0.4	21.6 ± 0.5	<0.001

The data are presented as marginal estimated mean ± s.e. FM, fat mass. Categories of cardiorespiratory fitness are low (first quartile), medium (second and third quartiles), or high (fourth quartile). Model 1: controlling for age and sex. Model 2: controlling for age, sex, and energy intake/weight. Model 3: controlling for age, sex, and carbohydrate/weight. Model 4: controlling for age, sex, and protein/weight. Model 5: controlling for age, sex, and fat/weight. All of the pairwise mean comparisons using the Bonferroni post-hoc test were statistically significant (low > medium > high, *p* < 0.05).

**Table 4:** ANCOVA models comparing mean differences in fat mass percentage by categories of dietary factors in children.

	Model 1				Model 6			
	Low	Medium	High	*p*-Value	Low	Medium	High	*p*-Value
EI/Weight Categories								
FM (%)	31.2 ± 0.6	25.6 ± 0.4	20.4 ± 0.6	<0.001	29.7 ± 0.5	25.7 ± 0.3	21.6 ± 0.5	<0.001
Carbohydrate/Weight Categories								
FM (%)	30.2 ± 0.6	25.8 ± 0.4	20.8 ± 0.6	<0.001	29.0 ± 0.5	25.7 ± 0.4	22.3 ± 0.5	<0.001
Protein/Weight Categories								
FM (%)	29.6 ± 0.6	25.5 ± 0.5	22.1 ± 0.6	<0.001	28.3 ± 0.5	25.6 ± 0.4	23.2 ± 0.5	<0.001
Fat/Weight Categories								
FM (%)	29.6 ± 0.6	26.0 ± 0.4	21.1 ± 0.6	<0.001	28.4 ± 0.5	26.2 ± 0.4	22.0 ± 0.5	<0.001

The data are presented as marginal estimated mean ± s.e. EI, energy intake; FM, fat mass. Categories of EI/weight, carbohydrate/weight, protein/weight, and fat/weight are low (first quartile), medium (second and third quartiles), or high (fourth quartile). Model 1: controlling for age and sex. Model 6: controlling for age, sex, and cardiorespiratory fitness. All of the pairwise mean comparisons using the Bonferroni post-hoc test were statistically significant (low > medium > high, *p* < 0.05).

## Supplementary Materials

The following are available online at http://www.mdpi.com/2072-6643/10/3/358/s1, Table S1: ANCOVA models comparing mean differences in body composition variables by cardiorespiratory fitness categories in children, Table S2: ANCOVA models comparing mean differences in body composition variables by categories of dietary factors in children, Figure S1: Simple mediation models of the relationship between dietary factors and body mass index, using CRF as a mediator, controlling for age, Figure S2: Simple mediation models of the relationship between dietary factors and waist circumference, using CRF as a mediator, controlling for age, Figure S3: Simple mediation models of the relationship between dietary factors and fat mass index, using CRF as a mediator, controlling for age.
