# Supplementary material for: Cardiorespiratory Fitness as a Mediator of the Influence of Diet on Obesity in Children
Source: Nutrients. 2018 Mar 16;10(3):358. doi: 10.3390/nu10030358 (PMC5872776; doi:10.3390/nu10030358)
Supplement: Supplementary file 1 [file nutrients-10-00358-s001.pdf]

Table S1. ANCOVA models comparing mean differences in body composition variables by cardiorespiratory fitness categories in children.

	Cardiorespiratory fitness categories																			
	Model 1				Model 2				Model 3				Model 4				Model 5			
	Low	Medium	High	p-value	Low	Medium	High	p-value	Low	Medium	High	p-value	Low	Medium	High	p-value	Low	Medium	High	p-value
BMI (kg/m^2)	22.9±0.4	19.3±0.2	16.9±0.3	<0.001	21.9±0.3	19.4±0.2	17.5±0.3	<0.001	22.1±0.3	19.3±0.2	17.5±0.3	<0.001	22.2±0.4	19.4±0.2	17.3±0.3	<0.001	22.2±0.4	19.4±0.2	17.2±0.3	<0.001
WC (cm)	77.5±1.0	68.9±0.6	62.3±0.9	<0.001	75.0±0.9	69.1±0.5	63.8±0.8	<0.001	75.5±0.9	68.9±0.5	63.7±0.8	<0.001	75.9±0.9	69.0±0.6	63.3±0.8	<0.001	75.9±0.9	69.1±0.6	63.1±0.8	<0.001
FMI (kg/m^2)	7.4±0.2	5.1±0.1	3.6±0.2	<0.001	6.9±0.2	5.2±0.1	4.0±0.2	<0.001	7.0±0.2	5.1±0.1	4.0±0.2	<0.001	7.1±0.2	5.1±0.1	3.9±0.2	<0.001	7.1±0.2	5.2±0.1	3.8±0.2	<0.001

The data are presented as marginal estimated mean±s.e. BMI, body mass index; WC, waist circumference; FMI, fat mass index. Categories of cardiorespiratory fitness are low (first quartile), medium (second and third quartiles), or high (fourth quartile).

Model 1: controlling for age and sex. Model 2: controlling for age, sex and energy intake/weight. Model 3: controlling for age, sex and carbohydrate/weight. Model 4: controlling for age, sex and protein/weight. Model 5: controlling for age, sex and fat/weight. All of the pairwise mean comparisons using the Bonferroni post-hoc test were statistically significant (low >medium >high, $p < 0.05$).

Table S2: ANCOVA models comparing mean differences in body composition variables by categories of dietary factors in children.

	Model 1				Model 6			
	Low	Medium	High	p-value	Low	Medium	High	p-value
EI/weight categories								
BMI kg/m²)	22.4±0.2	19.4±0.2	16.5±0.3	<0.001	21.6±0.3	19.4±0.2	17.1±0.3	<0.001
WC (cm)	76.6±0.9	68.6±0.6	61.7±0.9	<0.001	74.5±0.8	68.8±0.5	63.3±0.8	<0.001
FMI (kg/m²)	7.1±0.2	5.1±0.1	3.4±0.2	<0.001	6.6±0.2	5.1±0.1	3.9±0.2	<0.001
Carbohydrate/weight categories								
BMI (kg/m²)	22.2±0.3	19.4±0.2	16.6±0.3	<0.001	21.6±0.3	19.3±0.2	17.4±0.3	<0.001
WC (cm)	75.9±0.9	68.9±0.6	61.8±0.9	<0.001	74.3±0.8	68.7±0.5	63.8±0.8	<0.001
FMI (kg/m²)	6.9±0.2	5.2±0.2	3.5±0.2	<0.001	6.5±0.2	5.1±0.1	4.0±0.2	<0.001
Protein/weight categories								
BMI (kg/m²)	21.8±0.4	19.3±0.3	17.2±0.4	<0.001	21.2±0.3	19.3±0.2	17.8±0.3	<0.001
WC (cm)	75.0±1.0	68.3±0.7	63.8±0.9	<0.001	73.2±0.8	68.5±0.6	65.4±0.8	<0.001
FMI (kg/m²)	6.7±0.2	5.1±0.2	3.9±0.2	<0.001	6.2±0.2	5.1±0.1	4.3±0.2	<0.001
Fat/weight categories								
BMI (kg/m²)	21.7±0.4	19.5±0.3	17.0±0.4	<0.001	21.0±0.3	19.6±0.2	17.4±0.3	<0.001
WC (cm)	74.8±0.9	68.9±0.7	62.9±0.9	<0.001	73.1±0.8	69.2±0.6	64.0±0.8	<0.001
FMI (kg/m²)	6.6±0.2	5.3±0.2	3.7±0.2	<0.001	6.2±0.2	5.3±0.1	4.0±0.2	<0.001

The data are presented as marginal estimated mean±s.e. EI, energy intake; BMI, body mass index; WC, waist circumference; FMI, fat mass index. Categories of EI/weight, carbohydrate/weight, protein/weight and fat/weight are low (first quartile), medium (second and third quartiles), or high (fourth quartile).

Model 1: controlling for age and sex. Model 6: controlling for age, sex and cardiorespiratory fitness. All of the pairwise mean comparisons using the Bonferroni post-hoc test were statistically significant (low >medium >high, $p< 0.05$).

Figure S1. Simple mediation models of the relationship between dietary factors and body mass index, using CRF as a mediator, controlling for age.

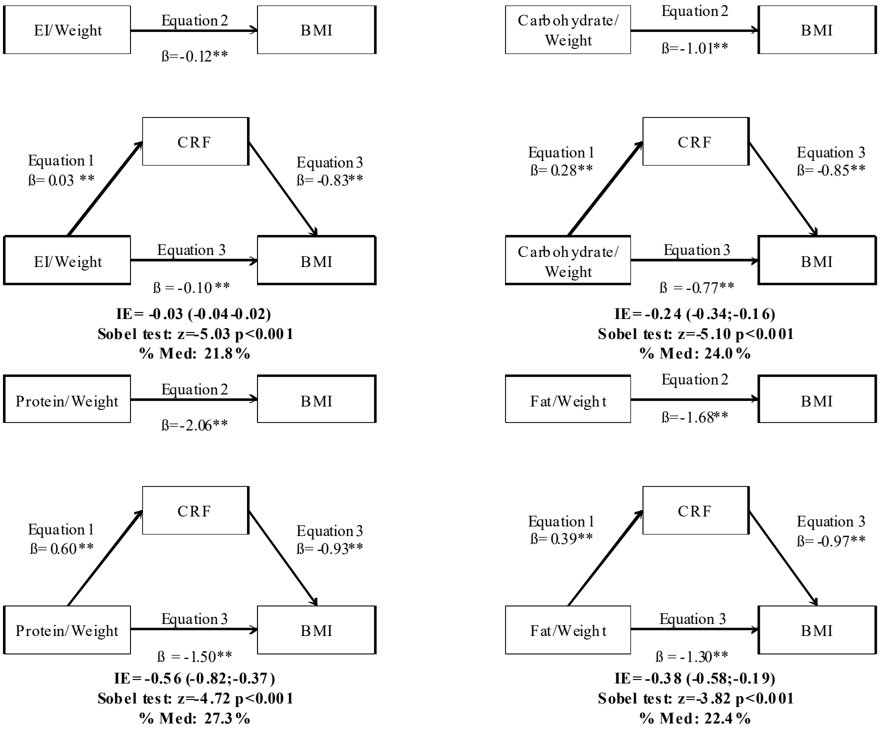

EI, energy intake; CRF, cardiorespiratory fitness; BMI, Body mass index; IE, indirect effect; %Med, percentage mediated by proposed mediator. **p< 0.001.

Figure S2. Simple mediation models of the relationship between dietary factors and waist circumference, using

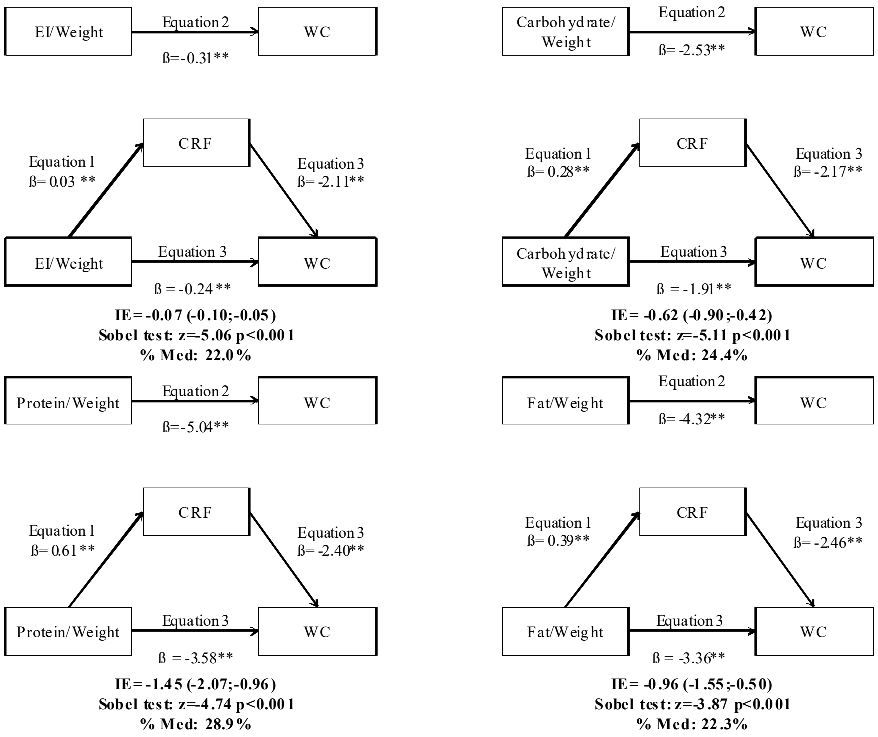

CRF as a mediator, controlling for age.

EI, energy intake; CRF, cardiorespiratory fitness;; WC, waist circumference; IE, indirect effect; %Med, percentage mediated by proposed mediator. **p< 0.001.

Figure S3. Simple mediation models of the relationship between dietary factors and fat mass index, using CRF

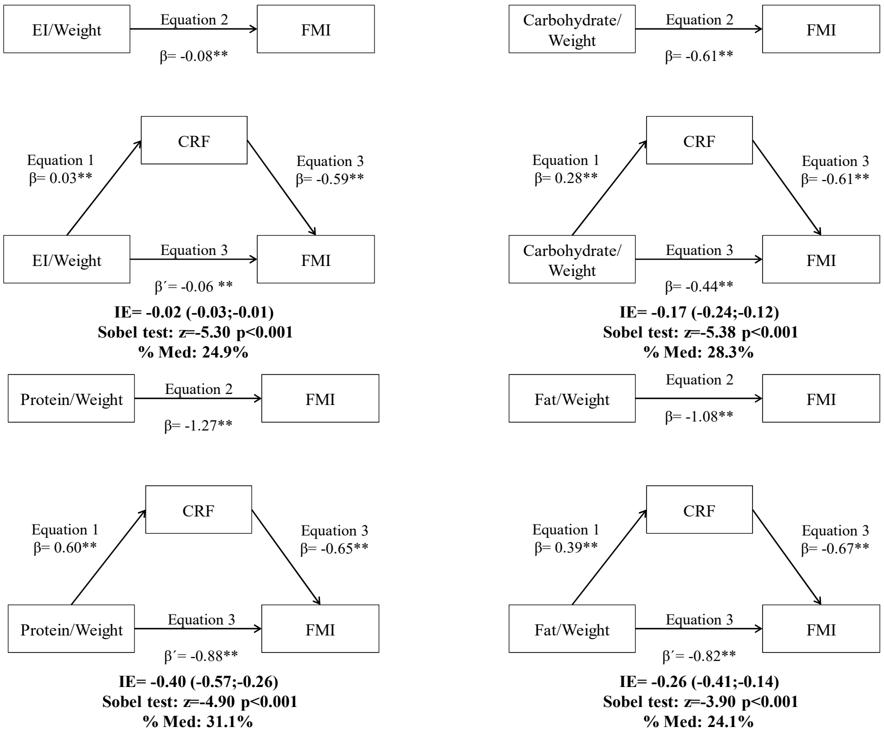

as a mediator, controlling for age.

EI, energy intake; CRF, cardiorespiratory fitness; FMI, fat mass index; IE, indirect effect; %Med, percentage mediated by proposed mediator. **p< 0.001.
